# Supplementary material for: Rates, patterns, and predictors of specialty palliative care consultation among patients with acute-on-chronic liver failure
Source: JHEP Rep. 2023 Nov 30;6(2):100976. doi: 10.1016/j.jhepr.2023.100976 (PMC10808910; doi:10.1016/j.jhepr.2023.100976)
Supplement: Multimedia component 1 [file mmc1.pdf]

Rates, patterns, and predictors of specialty palliative care consultation among patients with ACLF

Arpan Patel, Anne Walling, Fasiha Kanwal, Marina Serper, Ruben Hernaez, Vinay Sundaram, David Kaplan, Tamar Taddei, and Nadim Mahmud

Table of contents

Table S1……………………………………………………………………………………..2

Table S2……………………………………………………………………………………..3

Table S3……………………………………………………………………………………..3

Table S4……………………………………………………………………………………..3

Table S5……………………………………………………………………………………..3

Table S6……………………………………………………………………………………..5

Table S7……………………………………………………………………………………..5

Table S8……………………………………………………………………………………..6

Table S1 – Identification of Acute Decompensations (ADs) for EASL-CLIF ACLF

Decompensation	Classification		ICD-9/10 or CPT Codes
**Gastrointestinal Bleed***			
Algorithm: any of the following codes, in any position	UGIB – PUD		531.x, 532.x, 533.x, 534.x; K25.x, K26.x, K27.x, K28.x
	UGIB – non-PUD		456.0, 456.20, 535.x, 537.82, 537.83, 537.84, 578.0; I85.01, I85.11, K29.x, K31.819, K31.811, K31.82, K92.0
	Small bowel GIB		562.0x, 569.84, 569.85, 569.86; K57.1x, K55.20, K55.21, K63.81
	LGIB		562.1x, 569.3; K57.3x, K62.5
	GIB – NOS		578.1, 578.9; K92.1, K92.2
**Infection****			
Algorithm: any of the following codes, in any position	Bacteremia		038.x, 790.7; A40.x, A41.x
	SST/ MSK	Cellulitis	681.x, 682.x; L03.x, K12.2
		Septic arthritis	711.0x excluding 711.08; M00.x
		Osteomyelitis	730.0x, 730.1x, 730.2x; M86.x
	Spontaneous Bacterial Peritonitis		567.23; K65.2
	Pneumonia		481.x, 482.x; J13, J14, J15.x, J18.1, A48.1
	Pyelonephritis/UTI		590.x, 599.0; N10, N11.x, N12, N15.1, N15.9, N16, N28.84, N28.85, N28.86, N39.0
	Other	Endocarditis	421.x; I33.0, I33.9, I39
		Meningitis	320.x; G00.x, G01, G04.2
	Fungal Infection		112.5x, 117.3x, 995.91; B37.7x, B44.9x
**Hepatic Encephalopathy*****			
Algorithm: any of the following codes in any position PLUS lactulose OR rifaximin administered during inpatient admission (see supplemental table 4 for medication identification)	Hepatic encephalopathy		572.2; K72.91
	Viral hepatitis with hepatic coma		070.0, 070.2x, 070.40, 070.41, 070.44, 070.49, 070.6;<br>B15.0, B16.2, B19.11, B16.0, B17.11
**Ascites**			
Algorithm: any of the following codes, in any position	Ascites codes		789.51, 789.59; R18.x
	Paracentesis procedure codes		49080, 49081, 49082, 49083

** Adapted from Abraham et al., Wahl et al., and Siddiqui et al.*

*** Adapted from Schneeweiss et al. and Patkar et al.*

**** Adapted from Lo Re et al.*

Table S2 – Identification of Respiratory Organ Failure
Algorithm: presence of any of the following inpatient CPT codes

ICD-9/10, ICD-9/10-CM, or CPT Codes	Description
31500, 96.05	Endotracheal intubation procedure
94002, 94003, 94004, 94005, 94656, 94657, 94660, 94662, 96.7x, 5A1935Z, 5A1945Z, 5A1955Z	Mechanical ventilation and management
Z99.11	Ventilator dependent status

Table S3 – Identification of Brain Organ Failure
Algorithm: any of the hepatic encephalopathy ICD-9 codes in any position (see supplemental table 1) PLUS lactulose OR rifaximin administered within 28 days of inpatient admission. Identification of these medications was performed as follows:

Medication	SQL Query (VHA database)
Lactulose	WHERE medication LIKE '%LACTULO%' OR medication LIKE '%CONSTULO%' OR medication LIKE '%ENULO%'
Rifaximin	WHERE medication LIKE '%RIFAX%' OR medication LIKE '%XIFAX%'

**Obtained from the "BCMADispensedDrug" table in the VHA CDW; applied within 28 days of admission*

Table S4 – Identification of Inpatient Renal Replacement Therapy
Algorithm: presence of any of the following inpatient CPT codes

CPT Code	Description
90935, 90937	Hemodialysis
90945, 90947	Continuous renal replacement therapy

Table S5 – Identification of Vasopressors for Circulatory Failure

Vasopressors Classified	SQL Query (VHA database)
Norepinephrine<br>Epinephrine<br>Vasopressin	WHERE medication LIKE '%EPINEPHRI%' OR medication LIKE '%VASOPRESSI%' OR medication LIKE '%DOBUTAMIN%' OR

Dobutamine<br>Dopamine<br>Phenylephrine	medication LIKE ‘%LEVOPHED%’ OR medication LIKE ‘%DOPAMINE%’ OR medication LIKE ‘%PHENYLEPHRIN%’

** Obtained from the “iv_pharmacy_patient” table in the VHA CDW; applied within 28 days of admission*

Table S6 – Mixed-Effects Logistic Regression Models for Late Inpatient Palliative Care Consultation in 90-Day Mortality Subgroup*

Variable	OR (95% CI)	p-value
**Body Mass Index (per 5 unit change)**	1.09 (1.03-1.15)	0.001
**MELD-Na**	0.97 (0.96-0.98)	<0.001
**ACLF Grade**		
1	(ref)	
2	1.42 (1.19-1.68)	<0.001
3	2.07 (1.74-2.48)	<0.001
**Prior Palliative Care Consultation**	0.46 (0.39-0.55)	<0.001
**Hepatocellular Carcinoma**	0.58 (0.49-0.69)	<0.001
**High CIRCOM Score**	0.87 (0.76-0.99)	0.04
**Rural Center Location**	0.47 (0.25-0.88)	0.02

*Mixed effects models, designating VA facility as a random intercept, were used to generate odds ratios and p-values
**Level of significance: *p*-value <0.001
CIRCOM: cirrhosis comorbidity score

Table S7 – Mixed-Effects Logistic Regression Models for Inpatient Palliative Care Consultation, Evaluating the Potential Impact of ACLF Organ Failures*

Variable	OR (95% CI)	p-value
**Age (per year)**	1.03 (1.03-1.04)	<0.001
**Body Mass Index (per 5 unit change)**	0.93 (0.90-0.95)	<0.001
**MELD-Na**	1.01 (1.01-1.02)	<0.001
**Albumin (per 1 unit change)**	0.69 (0.65-0.73)	<0.001
**WBC Count (per 5 unit change)**	1.05 (1.02-1.08)	<0.001
**OF: Kidney**	1.69 (1.54-1.87)	<0.001
**OF: Liver**	2.47 (2.20-2.76)	<0.001
**OF: Coagulation**	1.26 (1.15-1.39)	<0.001
**OF: Brain**	1.36 (1.23-1.50)	<0.001
**OF: Circulatory**	1.82 (1.66-1.99)	<0.001
**Prior Palliative Care Consultation**	2.41 (2.17-2.69)	<0.001
**Hepatocellular Carcinoma**	2.18 (1.95-2.42)	<0.001
**High CIRCOM Score**	0.70 (0.64-0.76)	<0.001
**Cirrhosis Decompensation**	1.17 (1.08-1.27)	<0.001
**Prior TIPS**	0.66 (0.53-0.82)	<0.001

*Mixed effects models, designating VA facility as a random intercept, were used to generate odds ratios and p-values
**Level of significance: *p*-value <0.001
CIRCOM: cirrhosis comorbidity score

Table S8 – Mixed-Effects Logistic Regression Model with Individual CIRCOM Components and Any Cancer History Variable*

Variable	OR (95% CI)	p-value
Age (per year)	1.04 (1.03 - 1.04)	<0.001
Body Mass Index (per 5 unit change)	0.95 (0.92 - 0.98)	0.001
MELD-Na	1.02 (1.02 - 1.03)	<0.001
Albumin (per 1 unit change)	0.72 (0.67 - 0.77)	<0.001
WBC Count (per 5 unit change)	1.03 (1.00 – 1.07)	0.02
ACLF Grade		
1	(ref)	
2	1.74 (1.58 - 1.91)	<0.001
3	3.05 (2.73 - 3.41)	<0.001
Prior Palliative Care Consultation	2.62 (2.35 - 2.93)	<0.001
COPD	0.86 (0.79 - 0.94)	0.001
Chronic Kidney Disease	0.61 (0.56 - 0.66)	<0.001
Heart Failure	0.83 (0.76 - 0.91)	<0.001
Drug Abuse	1.01 (0.92 - 1.12)	0.78
Peripheral Artery Disease	0.82 (0.74 - 0.92)	<0.001
Epilepsy	0.88 (0.76 - 1.01)	0.08
History of Acute MI	1.19 (0.99 - 1.43)	0.06
Active MI	1.02 (0.88 - 1.18)	0.83
Any Cancer History	1.42 (1.31 - 1.54)	<0.001
Cirrhosis Decompensation	1.15 (1.06 - 1.25)	0.001
Prior TIPS	0.64 (0.52 - 0.80)	<0.001

*Mixed effects models, designating VA facility as a random intercept, were used to generate odds ratios and p-values
**Level of significance: *p*-value <0.001
CIRCOM: cirrhosis comorbidity score
