# Supplementary material for: Rates, patterns, and predictors of specialty palliative care consultation among patients with acute-on-chronic liver failure
Source: JHEP Rep. 2023 Nov 30;6(2):100976. doi: 10.1016/j.jhepr.2023.100976 (PMC10808910; doi:10.1016/j.jhepr.2023.100976)
Supplement: Multimedia component 4 [file mmc4.pdf]

Rates, patterns, and predictors of specialty palliative care consultation among patients with acute-on-chronic liver failure

Arpan Patel,[1,2,3,*] Anne Walling,[2,3] Fasiha Kanwal,[4,5] Marina Serper,[6,7,8] Ruben Hernaez,[4,5] Vinay Sundaram,[9] David Kaplan,[6,7] Tamar Taddei,[10,11] Nadim Mahmud[6,7,8,12]

[1]Vatche and Tamar Manoukian Division of Digestive Diseases, David Geffen School of Medicine at UCLA, Los Angeles, CA, United States; [2]Department of Medicine, Greater Los Angeles VA Healthcare System, Los Angeles, CA, United States; [3]Division of General Internal Medicine and Health Services Research, David Geffen School of Medicine at UCLA, Los Angeles, CA, United States; [4]Section of Gastroenterology and Hepatology, Michael E. DeBakey VA Medical Center and Baylor College of Medicine, Houston, TX, United States; [5]Department of Internal Medicine, Houston VA Health Services Research and Development Center for Innovations in Quality, Effectiveness and Safety, Michael E. DeBakey Veterans Affairs Medical Center, Houston, TX, United States; [6]Division of Gastroenterology and Hepatology, Perelman School of Medicine, University of Pennsylvania, Philadelphia, PA, United States; [7]Department of Medicine, Corporal Michael J. Crescenz VA Medical Center, Philadelphia, PA, United States; [8]Leonard David Institute of Health Economics, University of Pennsylvania Perelman School of Medicine, Philadelphia, PA, United States; [9]Karsh Division of Gastroenterology and Hepatology and Comprehensive Transplant Center, Cedars-Sinai Medical Center, Los Angeles, CA, United States; [10]Division of Digestive Diseases, Yale University School of Medicine, New Haven, CT, United States; [11]VA Connecticut Healthcare System, West Haven, CT, United States; [12]Center for Clinical Epidemiology and Biostatistics, Department of Biostatistics, Epidemiology & Informatics, Perelman School of Medicine, University of Pennsylvania, Philadelphia, PA, United States

JHEP Reports **2024.** https://doi.org/10.1016/j.jhepr.2023.100976

Background & Aims: There is growing acceptance that principles of palliative care should be integrated into the management of serious illnesses affecting the liver, such as acute-on-chronic liver failure (ACLF). However, rates, patterns, and predictors of specialty palliative care consultation among patients with ACLF have not been well-described.
Methods: We performed a retrospective cohort study of patients hospitalized with ACLF between 1/1/2008 and 12/31/2018 using the VOCAL cohort. Patients were followed until 6/2021. We used mixed-effects regression analyses to identify significant patient and facility factors associated with palliative care consultation. We examined timing of consultation, the influence of ACLF characteristics, and facility-level variation on receipt of palliative care consultation.
Results: We identified 21,987 patients hospitalized with ACLF, of whom 30.5% received specialty palliative care consultation. Higher ACLF grade (ACLF-2 [odds ratio (OR) 1.82, 95% CI 1.67-1.99], ACLF-3 [OR 3.06, 95% CI 2.76-3.40]), prior specialty palliative care consultation (OR 2.62, 95% CI 2.36-2.91), and hepatocellular carcinoma (OR 2.10, 95% CI 1.89-2.33) were associated with consultation. Consultation occurred latest and closest to the time of death for patients with ACLF-3 compared to ACLF-1 and ACLF-2. Significant facility-level variation in consultation persisted among patients with ACLF-3, despite adjusting for multiple patient and facility factors.
Conclusion: In this large cohort of hospitalized patients with ACLF, specialty palliative care consultation was rare, more common in patients with higher grade ACLF, and tended to occur closer to the time of death for the sickest patients. Greater attention should be placed on earlier integration of palliative care during acute hospitalizations in patients with ACLF.
Impact and implications: Though palliative care consultation is recommended for patients with acute-on-chronic liver failure, there is no data demonstrating how often this occurs during hospitalizations, on a population level. We found that consultation occurs in only 30.5% of patients and occurs later for patients with grade 3 acute-on-chronic liver failure. Our data should provoke clinicians to urgently consider quality improvement efforts to integrate palliative care into the management of these seriously ill patients.
Published by Elsevier B.V. on behalf of European Association for the Study of the Liver (EASL). This is an open access article under the CC BY license (http://creativecommons.org/licenses/by/4.0/).

Introduction

Acute-on-chronic liver failure (ACLF) is a clinical syndrome characterized by systemic inflammation, single or multiple organ failure, and a uniquely high risk of short-term death in patients with underlying chronic liver disease or cirrhosis.[1] Because over half of patients with ACLF are expected to die within 90 days of diagnosis,[2] timely access to high-quality, multidisciplinary care is critical. This care involves identifying and addressing underlying precipitants of ACLF, managing complications of organ failure, and expediting referral for liver transplantation in order to achieve optimal curative outcomes.[3,4] Unfortunately, death is still likely for many patients, owing to variable access to expertise in managing ACLF,[2] known patient barriers to liver transplantation,[5] and progression of underlying disease despite maximal medical management.[6] Within this context, there has

Keywords: palliative care; cirrhosis; acute-on-chronic liver failure; decompensation; end of life.
Received 17 March 2023; received in revised form 29 October 2023; accepted 13 November 2023; available online 30 November 2023
* Corresponding author. Address: 1100 Glendon Avenue, Suite 850, Room 810, Los Angeles, California 90024, United States.
E-mail address: ArpanPatel@mednet.ucla.edu (A. Patel).

been increasing interest in integrating principles of palliative care into the management of patients with ACLF.[6,7]

Palliative care is an approach that focuses on the quality of life of patients and their families through the prevention, assessment and relief of suffering, pain, and other problems using symptom management, psychosocial care, communication, and support for complex decision making as well as transitions of care.[8] The goals of palliative and disease-directed care are complementary, and both approaches can be provided concurrently. Integrating palliative care with curative care can help ensure that shared decisions are not only directed at improving disease control but also incorporating patient and caregivers' goals, values, and priorities in the face of uncertain outcomes, which are common for patients with ACLF.[9] Specialty palliative care consultation has been associated with increased patient and clinician communication about goals of care,[10] lower rates of life-sustaining treatments,[11] and reduced readmission for hospitalized adults with decompensated cirrhosis.[12,13] Though incorporation of specialty palliative care teams into the management of ACLF has been supported by recently developed clinical guidelines,[7] little is known regarding how often this occurs. The goal of this study was to describe patterns of specialty palliative care consultation, along with patient and facility factors associated with higher rates of consultation, in hospitalized adults with ACLF.

Patients and methods

Data source and cohort creation

We conducted a retrospective cohort study of patients with cirrhosis using data from the VOCAL (Veterans Outcomes and Costs Associated with Liver Disease) cohort in the Veterans Health Administration (VHA). Prior publications have detailed the derivation of this cohort, which has also been used for multiple studies on ACLF.[14–17] Patients with new diagnoses of cirrhosis were identified using a validated algorithm of one inpatient or two outpatient international classification of diseases, ninth and tenth revision (ICD-9/10) codes (571.2, 571.5, K74.6x, K70.3x)[18] between 1/1/2008 an 12/31/2018, and longitudinal data in this cohort were obtained through 6/1/2021. In this study, we included patients aged ≥18 years who were hospitalized with a diagnosis of ACLF of any grade (detailed below). For patients with multiple ACLF hospitalizations, we included only the first hospitalization. Patients were excluded if they had received liver transplantation prior to hospitalization.

ACLF hospitalizations were defined in accordance with the European Association for the Study of the Liver (EASL) criteria. This requires evidence of an acute decompensating event, such as infection, gastrointestinal bleed, ascites, or hepatic encephalopathy that is followed within 28 days by the development of multiple organ failures, which may include kidney failure, coagulation failure, liver failure, brain failure, respiratory failure, and/or circulatory failure. The highest grade was selected for inclusion.[19] The VHA dataset has been utilized in numerous prior studies for the ascertainment of EASL-ACLF criteria, owing to the granular nature of available data. Acute decompensating events and organ failures were classified using combinations of administrative codes, laboratory data, and medication administration data, as summarized in Tables S1-5.

Variables of interest

The primary outcome of interest was the presence of specialty palliative care consultation. This was ascertained from the VHA consult tables using structured query language (SQL) queries for completed specialty palliative care/hospice consultations, which could occur any time from hospital admission to discharge.[20] In a subgroup analysis, a secondary outcome of late palliative care consultation was explored. This was defined as any inpatient specialty palliative care consultation completed >50% of the duration into the total hospitalization. Early consultation was considered any consultation that occurred during the first half of the duration of the index hospitalization. Secondary outcomes included mortality, receipt of liver transplantation, and hospital length of stay. Short-term mortality at 28 and 90 days was ascertained from the Vital Status File.[14,21,22]

We grouped explanatory variables into patient and facility factors. For each patient at the time of hospitalization, we collected detailed data regarding demographics (age, sex, race), BMI, and comorbidities (history of diabetes, coronary artery disease, heart failure, atrial fibrillation). We also ascertained laboratory data (sodium, creatinine, albumin, total bilirubin, international normalized ratio, white blood cell count, aspartate aminotransferase, alanine aminotransferase, platelet count) and vital sign data in the first 24 h after hospitalization. Model for end-stage liver disease score-sodium (MELD-Na) was computed from this laboratory data. Cirrhosis decompensating events and hepatocellular carcinoma (HCC) diagnoses were ascertained using well-validated VHA algorithms.[20,22] The cirrhosis comorbidity score (CIRCOM) was also computed and classified as low (0, 1 + 0, or 1 + 1) *vs.* high (3 + 0, 3 + 1, 5 + 0, or 5 + 1), consistent with prior methods.[23] For one exploratory analysis, individual comorbidities used to calculate this score were treated as separate covariates, along with an "any cancer" variable to isolate the effect of cancer *vs.* non-cancer-related comorbidities on palliative care consultation. Using methods detailed below, palliative care consultation in the year prior to hospital admission was captured for each patient. As mentioned previously, ACLF characteristics such as organ failures and acute decompensating events were also treated as explanatory variables. ACLF grades were calculated based on the number and types of organ failures – from 1 (least severe) to 3 (most severe) – again consistent with EASL-ACLF definitions.[19] Facility-level factors were captured for each VHA center (n = 123), including rural setting (yes/no), academic affiliation (yes/no), and distance of the VHA center to the nearest VA or non-VA transplant center (computed in miles using previously published methods).[24]

Statistical analyses

Descriptive statistics

All explanatory variables (patient and facility factors) and outcomes were expressed as medians and 25th and 75th percentiles for continuous data and as counts and percentages for categorical data.

Variables associated with specialty palliative care consultation

To identify factors associated with inpatient specialty palliative care consultation, we first performed bivariate analyses using the Wilcoxon rank-sum and Chi-square tests, respectively. Next, to identify significant predictors of palliative care consultation adjusting for other variables, we used multivariable logistic regression. First, univariable analysis using LOWESS (locally weighted scatterplot smoothing) curves was performed to identify potential non-linearity in the relationship between continuous variables and the outcome.[25] Explanatory variables involved in final models demonstrated approximate linearity and

did not require variable transformation. Next, reverse stepwise selection was first used to identify a preliminary candidate model from all potential explanatory variables listed in Table 1 including ACLF grade. Several modified clinician-driven models were then created, where *a priori* variables thought to be potentially meaningful were reintroduced. The final model was selected based on a minimized value of the Bayesian information criterion. Given the possibility that the baseline likelihood of consultation could vary across VHA centers, we then created a mixed-effects logistic regression model where VHA center was designated as a random intercept. For the final mixed-effects model, we presented odds ratios (ORs) and 95% CIs for each exposure.

Temporal trends and timing of specialty palliative care consultation
To assess temporal trends in consultation, we plotted the proportion of inpatient hospitalizations during which patients received consultation, stratified by ACLF grade. Given approximate linearity in the observed data, we used linear regression to identify significant trends over time. The proportion of hospitalizations with and without consultation were stratified by ACLF grade, acute decompensations, and organ failures and were compared using the Cochran-Armitage test for trend (*i.e.*, exploring possible increased consultation with increasing ACLF grade). Finally, kernel density plots were created to visualize the timing of consultation in terms of (1) days from hospitalization and (2) days from consultation until death. Each plot was stratified by ACLF grade, with medians compared using the Kruskal-Wallis test.

Facility-level variation in specialty palliative care consultation
To visualize the center-level variation in average likelihood of consultation, we computed the posterior predicted probability for each center from the mixed-effects model and plotted these data along with 95% CIs. To evaluate whether center-level variation in consultation would vary by perceived acuity of hospitalization, we performed secondary subgroup analyses limited to (1) patients who experienced 90-day mortality and (2) patients with ACLF-3. As before, we computed posterior predicted probabilities for consultation at the center level derived from adjusted mixed-effects models. Coefficient plots were produced for the primary and secondary mixed-effects models. Next, to explore the potential association between specific organ failures and consultation, we constructed a mixed-effects logistic regression model where individual organ failures were evaluated rather than ACLF grade. Finally, in an exploratory analysis, we followed a similar modeling procedure to identify factors associated with late consultation (defined as consultation >50% into hospitalization) in the subgroup of patients who experienced 90-day mortality. For all analyses, an alpha level of 5% was used as the threshold for statistical significance.

This study received Institutional Review Board approval from the Michael J. Crescenz Philadelphia Veterans Affairs Medical Center. All data management and analyses were performed using SQL and STATA/BE 17.0 (College Station, TX).

Results

Demographics and clinical characteristics of cohort

From 2008-2018, 21,987 patients were hospitalized with ACLF. Patients were mostly male (97.6%) and White (55.8%). Alcohol-associated liver disease (40.0%) was the most common etiology of underlying liver disease; 59% of patients had decompensated cirrhosis and 9% had a diagnosis of HCC. Median MELD-Na within 24 h of hospitalization was 25 (IQR 21–30). ACLF-1, ACLF-2, and ACLF-3 were diagnosed in 56%, 26%, and 18% of patients, respectively. The average hospital length of stay was 7 days (IQR 3–14). Within 90 days of ACLF diagnosis, 8,938 patients (40.6%) died and 69 (0.3%) received a liver transplant.

Demographics and clinical factors associated with inpatient specialty palliative care consultation

During their hospitalizations, 6,723 patients (30.5%) received specialty palliative care consultation. These rates varied among patients who died within 90 days of ACLF diagnosis (55%), patients who survived (14%), and patients who survived and ultimately received LT (7.3%). Only 14.1% of patients had received consultation prior to hospitalization. Compared with White patients, Hispanic patients were more likely, whereas Black patients were less likely, to receive inpatient consultation. Patients with alcohol-associated liver disease, prior history of consultation, and greater burden of liver disease were also more likely to receive consultation. This latter group included patients with decompensated cirrhosis, higher MELD-Na, and HCC. By contrast, patients with cardio-metabolic comorbidities (diabetes, coronary artery disease, heart failure, atrial fibrillation) and higher CIRCOM scores were less likely to receive consultation, along with patients hospitalized at academic-affiliated or rural hospitals (Table 1). Specialty palliative care consultation was, overall, more common among patients with ACLF-3 (51%) and ACLF-2 (36%), compared to ACLF-1 (21%; p <0.001). Adjusting for other covariates, ACLF-2 (odds ratio [OR] 1.82, 95% CI 1.67-1.99) and ACLF-3 (OR 3.06, 95% CI 2.76-3.40) were associated with higher rates of consultation, compared to ACLF-1. Consultation was also higher among patients with history of prior consultation (OR 2.62, 95% CI 2.36-2.91), decompensated cirrhosis (OR 1.14, 95% CI 1.06-1.24), and HCC (OR 2.10, 95% CI 1.89-2.33). Though higher age, MELD-Na, and white blood cell count were associated with higher consultation in our final model, higher BMI, serum albumin, CIRCOM score, and academic center affiliation were associated with lower rates of consultation (Table 2). In an exploratory analysis, we found that when separating individual components of the CIRCOM score in our model, presence of any cancer diagnosis was associated with a higher rate of consultation, whereas non-cancer comorbidities were associated with lower rates of consultation (Table S8).

ACLF characteristics associated with specialty palliative care consultation

Infection (55.3%) and ascites (39.5%) were the most common acute decompensations, and kidney failure (76.2%) was the most common organ failure. Across all acute decompensations and organ failures, consultation was associated with higher ACLF grade (Table 3; each p <0.001). For example, of ACLF-1 patients with kidney organ failure, 21.0% received consultation, *vs.* 51.7% of ACLF-3 patients with kidney organ failure. Among patients with ACLF-3, certain decompensations and organ failures were associated with lower rates of consultation. A lower proportion of patients with ACLF-3 and gastrointestinal bleed received consultation relative to other acute decompensations (*e.g.*, 48.9% *vs.* 54.5% for ascites or 54.7% for hepatic encephalopathy). Patients with ACLF-3 experiencing respiratory failure (45.0%) or circulatory failure (47.6%) had a lower likelihood of receiving consultation, relative to other organ failures, whereas those with

liver organ failure had the highest likelihood (60.1%). In the full ACLF cohort, when evaluating specific organ failures rather than ACLF severity grade in models, we found a positive association between presence of organ failures and consultation, with the exception of respiratory failure (Table S7).

Temporal trends and timing of specialty palliative care consultation

Rates of specialty palliative care consultation during ACLF hospitalizations increased steadily from 2008 to 2020 across all ACLF grades (ACLF-1 [β = 0.004, p = 0.001], ACLF-2 [β = 0.013, p <0.001], and ACLF-3 [β = 0.014, p = 0.016]) (Fig. 1). The median time to consultation was shortest in patients with ACLF-1, whereas consultation tended to occur later in patients with ACLF-2 or ACLF-3 (*e.g.*, median time to consultation 6 days for ACLF-3 *vs.* 3 days for ACLF-1, p <0.001; Fig. 2A). Consultations for ACLF-2 were evenly distributed across the duration of hospitalizations, while consultations for ACLF-3 tended to occur near the very end of hospitalizations (Fig. 2B). Consultation occurred closest to time of death for patients with ACLF-3 compared to ACLF-1 and ACLF-2 (*e.g.*, median time from consultation to death 6 days for ACLF-3 *vs.* 21 days for ACLF-2, p <0.001; Fig. 2C). In an exploratory analysis of patients with consultation who died within 90 days, a total of 2,395 (49.4%) patients received late consultation (in the latter 50% of the hospitalization). Variables associated with late consultation included ACLF-2 (OR 1.42, 95% CI 1.19-1.68) and ACLF-3 (OR 2.08, 95% CI 1.74-2.48) relative to ACLF-1, whereas prior consultation, HCC, higher CIRCOM score, and higher MELD-Na score were associated with earlier consultation (Table S6).

Table 1. Hospitalized ACLF cohort characteristics, stratified by inpatient specialty palliative care consultation status.

Factor	No SPC consultation (n = 15,264)	SPC consultation (n = 6,723)	*p* value*
Age, median (interquartile range [IQR])	62.4 (57.2, 67.7)	62.8 (57.7, 68.6)	<0.001
Male sex	14,907 (97.7%)	6,558 (97.5%)	0.60
Race			<0.001
White	8,514 (55.8%)	3,753 (55.8%)	
Black	3,443 (22.6%)	1,316 (19.6%)	
Hispanic	1,297 (8.5%)	678 (10.1%)	
Asian	208 (1.4%)	69 (1.0%)	
Other	1,802 (11.8%)	907 (13.5%)	
BMI, median (IQR)	29.1 (25.4, 33.8)	28.2 (24.5, 32.5)	<0.001
Etiology of liver disease			<0.001
Hepatitis C	1,960 (12.8%)	820 (12.2%)	
Hepatitis B	132 (0.9%)	51 (0.8%)	
Alcohol-associated liver disease	5,952 (39.0%)	2,791 (41.5%)	
HCV+ALD	3,761 (24.6%)	1,653 (24.6%)	
Non-alcoholic fatty liver disease	3,158 (20.7%)	1,256 (18.7%)	
Other	301 (2.0%)	152 (2.3%)	
CIRCOM score			<0.001
Low	3,693 (24.2%)	2,555 (38.0%)	
High	11,571 (75.8%)	4,168 (62.0%)	
Diabetes mellitus	11,021 (72.2%)	4,197 (62.4%)	<0.001
Coronary artery disease	5,967 (39.1%)	2,124 (31.6%)	<0.001
Heart failure	5,503 (36.1%)	1,887 (28.1%)	<0.001
Atrial fibrillation	3,298 (21.6%)	1,257 (18.7%)	<0.001
Hepatocellular carcinoma	1,372 (9.0%)	1,390 (20.7%)	<0.001
Prior history of decompensated cirrhosis	9,051 (59.3%)	4,405 (65.5%)	<0.001
TIPS	588 (3.9%)	175 (2.6%)	<0.001
Prior PC consultation	1,524 (10.0%)	1,568 (23.3%)	<0.001
Sodium, median (IQR)	134 (131–137)	133 (128–136)	<0.001
Creatinine, median (IQR)	2.2 (1.6–3.2)	2.06 (1.4–3.005)	<0.001
AST, median (IQR)	48 (29–88)	69 (38–132)	<0.001
ALT, median (IQR)	30 (19–49)	36 (22–63)	<0.001
Albumin, median (IQR)	2.6 (2.1–3.1)	2.4 (2–2.8)	<0.001
Total bilirubin, median (IQR)	1.6 (0.8–3.6)	3 (1.31–7.6)	<0.001
INR, median (IQR)	1.5 (1.2–2.06)	1.7 (1.39–2.3)	<0.001
PLT count, median (IQR)	114 (73–170)	105 (66–162)	<0.001
WBC count, median (IQR)	7.74 (5.36–11.5)	9 (6.1–13.4)	<0.001
MELD, median (IQR)	22 (18–27)	24 (19–30)	<0.001
MELD-Na, median (IQR)	24 (20–29)	27 (22–32)	<0.001
Max temp, median (IQR)	98.4 (97.9–98.9)	98.2 (97.8–98.8)	<0.001
Min temp, median (IQR)	97.4 (96.9–98)	97.3 (96.8–97.8)	<0.001
Max HR, median (IQR)	88 (78–96)	91 (81–97)	<0.001
Max RR, median (IQR)	20 (18–21)	20 (18–22)	<0.001
Academic-affiliated hospital	9,993 (65.5%)	4226 (62.9%)	<0.001
Distance to transplant center (miles), median (IQR)	3.4 (0.6–24.5)	3.5 (0.7–39.4)	<0.001
Rural hospital	604 (4.0%)	235 (3.5%)	0.09

ACLF, acute-on-chronic liver failure; ALD, alcohol-associated liver disease; ALT, alanine aminotransferase; AST, aspartate aminotransferase; CIRCOM, cirrhosis comorbidity score; HR, heart rate; INR, international normalized ratio; MELD(-Na), model for end-stage liver disease(-sodium); PC, palliative care; RR, respiratory rate; SPC, specialty palliative care; TIPS, transjugular intrahepatic portosystemic shunt; WBC, white blood cell.
* Wilcoxon rank-sum and Chi-square tests were used to determine statistically significant differences; level of significance: p = 0.001.

Table 2. Predictors of inpatient specialty palliative care consultation.

Variable	Original model*		90-day mortality subcohort*		ACLF-3 subcohort*	
	OR (95% CI)	p value**	OR (95% CI)	p value**	OR (95% CI)	p value**
Age (per year)	1.03 (1.03-1.04)	<0.001	1.01 (1.01-1.02)	<0.001	1.01 (1.01-1.02)	<0.001
Male sex	–	–	–	–	0.66 (0.45-0.96)	0.03
BMI (per 5 unit change)	0.93 (0.90-0.95)	<0.001	0.95 (0.92-0.98)	0.005	0.95 (0.90-1.00)	0.04
MELD-Na	1.02 (1.02-1.03)	<0.001	–	–	–	–
Albumin (per 1 unit change)	0.69 (0.65-0.74)	<0.001	–	–	–	–
WBC count (per 5 unit change)	1.05 (1.02-1.08)	0.001	1.04 (1.01-1.08)	0.02	–	–
ACLF grade						
1	(ref)		(ref)		–	–
2	1.83 (1.67-2.00)	<0.001	1.49 (1.33-1.68)	<0.001	–	–
3	3.10 (2.79-3.44)	<0.001	1.56 (1.39-1.75)	<0.001	–	–
Prior palliative care consultation	2.44 (2.19-2.72)	<0.001	1.43 (1.26-1.62)	<0.001	1.51 (1.23-1.86)	<0.001
Hepatocellular carcinoma	2.21 (1.99-2.46)	<0.001	1.78 (1.57-2.02)	<0.001	1.32 (1.09-1.60)	0.005
High CIRCOM score	0.72 (0.66-0.78)	<0.001	0.95 (0.86-1.05)	0.30	0.79 (0.69-0.91)	0.001
Prior history of decompensated cirrhosis	1.15 (1.06-1.24)	0.001	–	–	–	–
Prior TIPS	0.64 (0.52-0.79)	<0.001	–	–	–	–

ACLF, acute-on-chronic liver failure; CIRCOM, cirrhosis comorbidity score; MELD-Na, model for end-stage liver disease-sodium; TIPS, transjugular intrahepatic portosystemic shunt; WBC, white blood cell.
* Mixed-effects models, designating Veterans Affairs facility as a random intercept, were used to generate odds ratios and *p* values.
** Level of significance: *p* value <0.001.

Table 3. ACLF characteristics, stratified by ACLF grade and inpatient specialty palliative care consultation status*.

Factor	ACLF-1		ACLF-2		ACLF-3		p value†
	No SPC (n = 9,660)	SPC (n = 2,646)	No SPC (n = 3,692)	SPC (n = 2,094)	No SPC (n = 1,912)	SPC (n = 1,983)	
**Acute decompensation**							
Infection	4,891 (78.1%)	1,368 (21.9%)	1,884 (60.1%)	1,253 (39.9%)	1,333 (48.2%)	1,433 (51.8%)	<0.001
Gastrointestinal bleed	2,414 (80.6%)	582 (19.4%)	913 (63.2%)	531 (36.8%)	612 (51.0%)	587 (49.0%)	<0.001
Ascites	3,509 (71.6%)	1,395 (28.5%)	1,310 (58.6%)	925 (41.4%)	700 (45.5%)	838 (54.5%)	<0.001
Hepatic encephalopathy	982 (79.8%)	248 (20.2%)	1,367 (69.1%)	611 (30.9%)	604 (45.4%)	726 (54.6%)	<0.001
**Organ failure**							
Kidney failure	7,734 (79.0%)	2,059 (21.0%)	2,245 (61.6%)	1398 (38.4%)	1,603 (48.3%)	1,717 (51.7%)	<0.001
Liver failure	111 (56.6%)	85 (43.4%)	596 (55.2%)	484 (44.8%)	638 (39.9%)	961 (60.1%)	<0.001
Coagulation failure	561 (79.9%)	141 (20.1%)	1,452 (67.5%)	699 (32.5%)	1,249 (46.8%)	1,420 (53.2%)	<0.001
Brain failure	982 (79.8%)	248 (20.2%)	1,367 (69.1%)	611 (30.9%)	604 (45.4%)	726 (54.6%)	<0.001
Respiratory failure	92 (68.7%)	42 (31.3%)	755 (66.2%)	386 (33.8%)	1,194 (55.0%)	977 (45.0%)	<0.001
Circulatory failure	180 (71.7%)	71 (28.3%)	969 (61.4%)	610 (38.6%)	1,463 (52.4%)	1,328 (47.6%)	<0.001

ACLF, acute-on-chronic liver failure; SPC, specialty palliative care.
† *p* values correspond to a Cochran-Armitage test for trend in the proportion of patients receiving inpatient palliative care consultation across increasing ACLF grades. Level of significance: *p* value <0.001.
* Percentages shown represent row percentages within each ACLF grade.

Facility-level variation in specialty palliative care consultation

Using posterior predicted probabilities calculated from our adjusted mixed-effects model, we found significant variation in rates of consultation by VHA facility (n = 123), ranging from 35-66% (Fig. 3A). In a subgroup analysis limited to patients who experienced 90-day mortality, there was far less adjusted center-level variation in consultation in these patients (*e.g.*, all centers essentially within +/- 10% probability of consultation; Fig. 3B), though predictors of consultation were similar (Table 2). In a subgroup analysis limited to patients with ACLF-3 (Table 2), the adjusted center-level variation in probability of consultation was similar to the primary (complete cohort) model (Fig. 3C).

Discussion

There is growing acceptance that palliative care should be offered to all patients with serious illnesses affecting the liver,[26,27] which extends to management of patients with ACLF.[7] However, our study found that less than one-third of hospitalized Veterans with ACLF received specialty palliative care consultation. Consultations increased steadily during the study period and were most common among patients with higher ACLF grades, who arguably have the highest palliative care needs,[28,29] as well as in those with history of prior consultation, HCC, and more advanced liver disease. However, patients with higher ACLF grades were also more likely to receive consultation later during their hospital course. Patients with non-cancer comorbidities and those who survived hospitalization were the least likely to receive any consultation. Collectively, our data suggest that clinician teams managing ACLF largely still perceive palliative care as end-of-life care.

Specialty palliative care consultation in our cohort was uncommon – far below rates observed in hospitalized Veterans with other serious illnesses, such as advanced cancer (73.5%), heart failure (46.7%) and end-stage renal disease (50.4%) nearing the end of life.[30]

Rates of consultation in this cohort, however, were higher than those observed in a national cohort of hospitalized adults with decompensated cirrhosis (4.5%)[31] and very similar to those

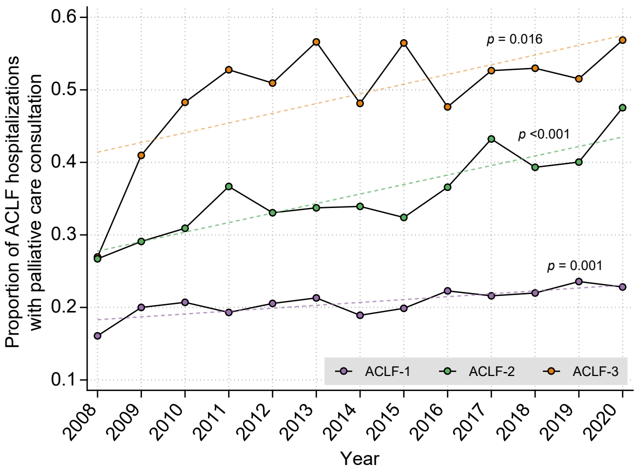

Fig. 1. Proportion of ACLF hospitalizations with specialty palliative care consultation over time. *Cochran-Armitage test was used for trend analysis. p <0.01 was considered statistically significant. ACLF, acute-on-chronic liver failure.

observed in patients with decompensated cirrhosis at the end of life (30.3%).[11] In these studies, specialty palliative care consultation was strongly influenced by patient race and facility factors, such as presence of academic teaching, urban or rural status, and hospital bed size. In our cohort, neither patient race nor facility factors were strongly associated with specialty palliative care consultation, reflecting more uniform access to these services across all VHA facilities.[32] The fact that rates of palliative care consultation increased in patients with ACLF over time aligns with the experience of multiple populations seen within and outside the VA.[33 34 35]

Higher ACLF grade was associated with a higher likelihood of consultation, but consultations occurred later and closer to end of life, mirroring observations from single-center studies of hospitalized adults with decompensated cirrhosis.[36,37] Clinicians treating patients with ACLF, such as hepatologists, may perceive palliative care to be synonymous with end-of-life care;[38–40] thus, palliative care consultations may be delayed until certain disease-directed treatments, including liver transplantation, are largely perceived as futile. This optimism carried by hepatologists regarding addressing conditions they perceive as reversible and keeping transplantation as an available option, however remote the possibility, may help explain why patients with ACLF-3 and gastrointestinal hemorrhage, respiratory failure, or circulatory failure were less likely to receive consultation.[41,42] It may also help explain why patients with higher BMI tended to receive later consultation and patients with lower albumin were more likely to receive consultation, as clinicians may have held off consultation until patients were overtly frail and exhibiting markers of malnutrition. Meanwhile, early palliative care consultation is associated with higher rates of advance care planning and more days spent alive outside the hospital, which are benefits that patients can experience irrespective of whether curative treatments remain a possibility.[11 43] Lastly, our findings indicate that rates of consultation may reflect institutional or cultural norms. For instance, we found marked variation in specialty palliative care consultation across facilities, which persisted for patients with ACLF-3, but not among patients who ultimately died, despite adjusting for key covariates. We also found that the presence of any cancer, compared to a non-cancer comorbidity, strongly influenced rates of consultation. Such a pattern has similarly been observed in a previous VA study,[30] which showed that patients with cancer tend to receive better access to palliative care than those with other serious illnesses. This may be due to traditional non-integration between palliative care services and non-cancer teams, along with confusion about whose role it is to deliver palliative care.[43] Developing shared mental models between specialty palliative care, hepatology, internal medicine, and other specialty teams that outline specific processes of care (such as conditions of referral) and standardize communication is an initial step towards promoting high-quality, multidisciplinary care for patients with ACLF, which is the ultimate goal.[7,9] Adoption of new guidelines for ACLF and palliative care in decompensated cirrhosis, which suggest earlier integration of palliative care teams,[7,44] may help accelerate shifts in these perceptions over time.

Other patient factors were associated with earlier rates of specialty palliative care consultation during hospitalizations,

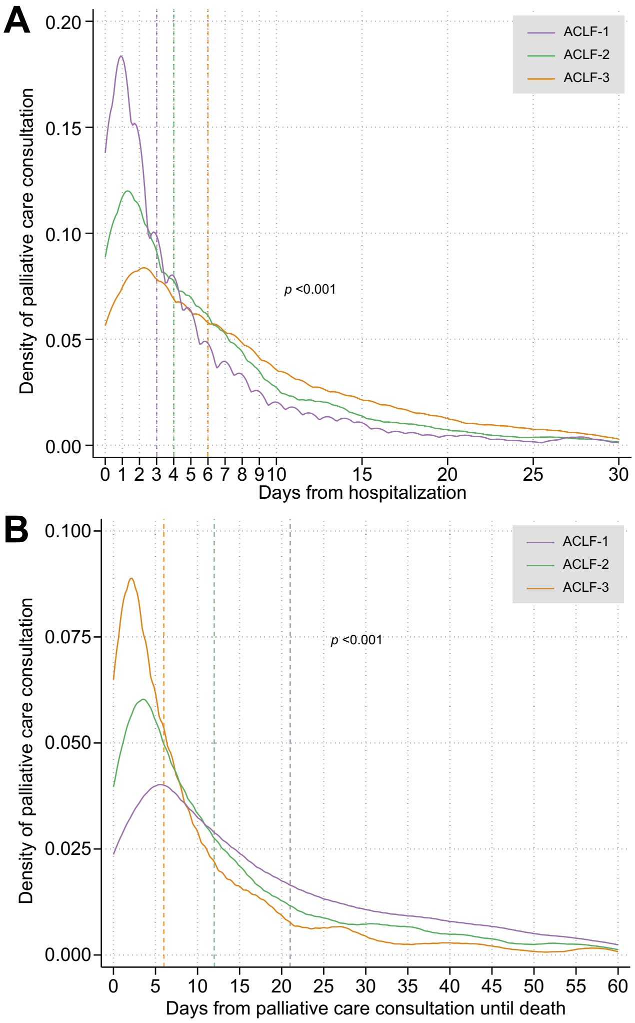

Fig. 2. Temporal distribution of inpatient specialty palliative care consultation, stratified by ACLF grade. Kernel density plots were created to visualize the timing of consultation in terms of (A) days from hospitalization and (B) days from consultation until death. Each plot was stratified by ACLF grade, with medians compared using the Kruskal-Wallis test. *Vertical hash lines correspond to median values for each ACLF grade. ACLF, acute-on-chronic liver failure.

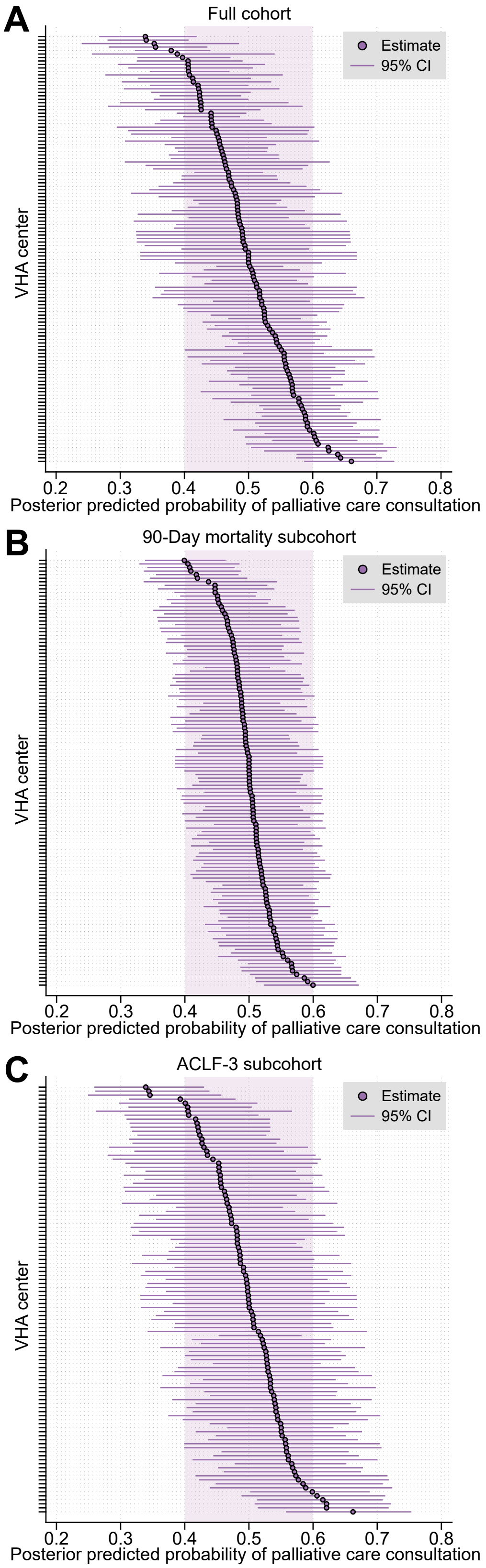

Fig. 3. Center variation in adjusted probability of specialty palliative care consultation in ACLF hospitalizations. * (A) In full cohort, (B) 90-day mortality subcohort, and (C) ACLF-3 subcohort. *Derived from mixed-effects logistic regression models in Table 3. ACLF, acute-on-chronic liver failure; VHA, Veterans Health Administration.

which are linked to more advance care planning and lower provision of life-sustaining treatments in patients with decompensated cirrhosis.[10,45] History of prior consultation and HCC facilitated greater and earlier access to consultation, reflecting the integrated nature of palliative care services within VA,[32] as well as generally greater acceptance of early palliative care among clinicians managing patients with advanced cancer.[27] Higher CIRCOM scores were also associated with earlier consultation, even though overall rates of consultation were lower. More qualitative studies are needed to better capture clinician perspectives when deciding or not deciding to consult specialty palliative care in specific clinical contexts during acute hospitalizations.

Our results should be interpreted in the context of its limitations. As is the case with many observational studies, it is possible that our exposures and outcomes were misclassified; however, wherever possible, we used validated algorithms to generate our key variables and only included patients followed actively within the VHA system. Selected ACLF organ failures, such as respiratory failure, may have been misclassified due to lack of fraction of inspired oxygen data, though in general the granularity of data afforded in the VHA is a major strength in classifying ACLF relative to national registry data.[46] Second, there is also the potential for bias due to residual confounding, but we believe our use of multiple different models and careful application of sensitivity analyses ensure that our findings are reliable. It is important to acknowledge that presence of specialty palliative care consultation is not synonymous with receipt of high-quality, goal-concordant care; however, there is strong evidence to suggest that consultation is associated with the achievement of several patient-centered outcomes.[47] Future work should consider evaluating for the presence of other palliative care process and outcome measures, including presence of advance care planning and family end-of-life satisfaction. Lastly, there are limitations in how generalizable our findings are to settings outside of the VHA, given that the patient population is predominantly male and has less access to liver transplantation compared to the general population. This may, for example, prevent us from understanding whether certain disparities based on gender and transplant consideration may exist for ACLF. Nonetheless, our data strongly justifies increasing access to palliative care for patients with ACLF within VHA. Quality improvement teams[48,49] in VHA that facilitate access to palliative care, standardize advance care planning documentation, and offer serious illness communication training to clinicians should consider prioritizing patients with ACLF and decompensated cirrhosis as populations with high needs. This may help reduce the variation in palliative care consultation seen across different facilities. Greater interest in this work across the VHA may ultimately lead to innovative models for the care of patients with cirrhosis that integrate early palliative care with disease-directed care.[50]

In conclusion, our study found that only 30% of patients hospitalized for ACLF received specialty palliative care. When performed, referrals often occurred near the end of life for patients with ACLF-2 and ACLF-3. This likely reflects the misperception that palliative care is synonymous with end-of-life care or medical futility. Earlier referrals occurred in patients with HCC, prior specialty palliative care consultation, and higher comorbidity. Quality improvement efforts through VHA have the potential to maximize multidisciplinary support and care in both outpatient and inpatient settings, which is critical in ensuring the achievement of patient-centered outcomes for this vulnerable population.

Abbreviations

ACLF, acute-on-chronic liver failure; EASL, European Association for the Study of the Liver; OR, odds ratio; VHA, Veterans Health Administration.

Financial support

Arpan Patel is supported by the National Institute of Alcohol Abuse and Alcoholism (P50-AA011999-23) and a VA internal grant (CSHIIP SWIFT Program). Nadim Mahmud is supported by the National Institute of Diabetes and Digestive and Kidney Diseases (K08-DK124577). Marina Serper is supported by a National Institutes of Health K23 grant (DK115897-03). David E. Kaplan has received support from Gilead, Glycotest and Bayer unrelated to the topic of this manuscript. He is also supported by VA Merit Grants (I01-CX-001933, I01-CX-002010). Tamar H. Taddei is supported by a VA Merit Grant (I01-CX-002010) and by the National Cancer Institute R01 (CA206465). Fasiha Kanwal is supported by National Cancer Institute (NCI U01 CA230999, and R01CA186566), Cancer Prevention & Research Institute of Texas grant (RP150587), VA Merit Grant (IIR 21-230-2) and is an investigator at the Veterans Administration Center for Innovations in Quality, Effectiveness and Safety (CIN 13-413), Michael E. DeBakey VA Medical Center, Houston, Texas.

Conflict of interest

The authors of this manuscript have no conflicts of interest to disclose.

Please refer to the accompanying ICMJE disclosure forms for further details.

Authors' contributions

Concept and design: Patel, Walling, Serper, Kaplan, Taddei, Mahmud. Acquisition, analysis, or interpretation of data: All authors. Drafting of the manuscript: Patel and Mahmud. Critical revision of the manuscript: All authors. Statistical analysis: Mahmud.

Obtained funding: none. Administrative, technical, or material support: Serper, Kaplan, Taddei, Mahmud. Supervision: Walling, Kanwal, Serper, Hernaez, Sundaram, Kaplan, Taddei, Mahmud.

Data availability statement

The data that support the findings of this study are available from the last author, NM. The data are not publicly available due to the fact that data contains information that could compromise the privacy of research participants.

Supplementary data

Supplementary data to this article can be found online at. https://doi.org/10.1016/j.jhepr.2023.100976.

References

[1] Arroyo V, Moreau R, Jalan R. Acute-on-Chronic liver failure. N Engl J Med May 28 2020;382(22):2137–2145. https://doi.org/10.1056/NEJMra1914900.
[2] Hernaez R, Kramer JR, Liu Y, et al. Prevalence and short-term mortality of acute-on-chronic liver failure: a national cohort study from the USA. J Hepatol Apr 2019;70(4):639–647. https://doi.org/10.1016/j.jhep.2018.12.018.
[3] Arroyo V. Acute-on-Chronic liver failure in cirrhosis requires expedited decisions for liver transplantation. Gastroenterol Apr 2019;156(5):1248–1249. https://doi.org/10.1053/j.gastro.2019.03.004.
[4] Sundaram V, Jalan R, Wu T, et al. Factors associated with survival of patients with severe acute-on-chronic liver failure before and after liver transplantation. Gastroenterology Apr 2019;156(5):1381–1391 e3. https://doi.org/10.1053/j.gastro.2018.12.007.
[5] Kanwal F, Hernaez R, Liu Y, et al. Factors associated with access to and receipt of liver transplantation in veterans with end-stage liver disease. JAMA Intern Med May 24 2021. https://doi.org/10.1001/jamainternmed.2021.2051.
[6] Engelmann C, Thomsen KL, Zakeri N, et al. Validation of CLIF-C ACLF score to define a threshold for futility of intensive care support for patients with acute-on-chronic liver failure. Crit Care Oct 10 2018;22(1):254. https://doi.org/10.1186/s13054-018-2156-0.
[7] Bajaj JS, O'Leary JG, Lai JC, et al. Acute-on-Chronic liver failure clinical guidelines. Am J Gastroenterol 2022;117(2):225–252.
[8] Sepulveda C, Marlin A, Yoshida T, et al. Palliative care: the world health organization's global perspective. J Pain Symptom Manage Aug 2002;24(2):91–96. https://doi.org/10.1016/s0885-3924(02)00440-2.
[9] Naik AD, Arney J, Clark JA, et al. Integrated model for patient-centered advanced liver disease care. Clin Gastroenterol Hepatol Jul 26 2019. https://doi.org/10.1016/j.cgh.2019.07.043.
[10] Lamba S, Murphy P, McVicker S, et al. Changing end-of-life care practice for liver transplant service patients: structured palliative care intervention in the surgical intensive care unit. J Pain Symptom Manage Oct 2012;44(4):508–519. https://doi.org/10.1016/j.jpainsymman.2011.10.018.
[11] Patel AA, Walling AM, Ricks-Oddie J, et al. Palliative care and health care utilization for patients with end-stage liver disease at the end of life. Clin Gastroenterol Hepatol Oct 2017;15(10):1612–1619 e4. https://doi.org/10.1016/j.cgh.2017.01.030.
[12] Shinall Jr MC, Karlekar M, Martin S, et al. COMPASS: a pilot trial of an early palliative care intervention for patients with end-stage liver disease. J Pain Symptom Manage Oct 2019;58(4):614–622. https://doi.org/10.1016/j.jpainsymman.2019.06.023.
[13] Adejumo AC, Kim D, Iqbal U, et al. Suboptimal use of inpatient palliative care consultation may lead to higher readmissions and Costs in end-stage liver disease. J Palliat Med Jan 2020;23(1):97–106. https://doi.org/10.1089/jpm.2019.0100.
[14] Kaplan DE, Dai F, Aytaman A, et al. Development and performance of an algorithm to estimate the child-turcotte-pugh score from a national electronic Healthcare database. Clin Gastroenterol Hepatol Dec 2015;13(13):2333–23341. https://doi.org/10.1016/j.cgh.2015.07.010. e1-6.
[15] Mahmud N, Kaplan DE, Taddei TH, et al. Incidence and mortality of acute-on-chronic liver failure using two definitions in patients with compensated cirrhosis. Hepatology May 2019;69(5):2150–2163. https://doi.org/10.1002/hep.30494.
[16] Mahmud N, Sundaram V, Kaplan DE, et al. Grade 1 acute on chronic liver failure is a predictor for subsequent grade 3 failure. Hepatol Jul 2020;72(1):230–239. https://doi.org/10.1002/hep.31012.
[17] Mahmud N, Chapin S, Goldberg DS, et al. Statin exposure is associated with reduced development of acute on chronic liver failure in a veterans affairs cohort. J Hepatol Jan 20 2022. https://doi.org/10.1016/j.jhep.2021.12.034.
[18] Kramer JR, Davila JA, Miller ED, et al. The validity of viral hepatitis and chronic liver disease diagnoses in Veterans Affairs administrative databases. Aliment Pharmacol Ther Feb 1 2008;27(3):274–282. https://doi.org/10.1111/j.1365-2036.2007.03572.x.
[19] Moreau R, Jalan R, Gines P, et al. Acute-on-chronic liver failure is a distinct syndrome that develops in patients with acute decompensation of cirrhosis. Gastroenterology Jun 2013;144(7):1426–1437. https://doi.org/10.1053/j.gastro.2013.02.042. 1437 e1-9.
[20] Serper M, Taddei TH, Mehta R, et al. Association of provider specialty and multidisciplinary care with hepatocellular carcinoma treatment and mortality. Gastroenterology Jun 2017;152(8):1954–1964. https://doi.org/10.1053/j.gastro.2017.02.040.
[21] Sohn MW, Arnold N, Maynard C, et al. Accuracy and completeness of mortality data in the department of veterans affairs. Popul Health Metr Apr 10 2006;4:2. https://doi.org/10.1186/1478-7954-4-2.
[22] Lo Re 3rd V, Lim JK, Goetz MB, et al. Validity of diagnostic codes and liver-related laboratory abnormalities to identify hepatic decompensation events in the Veterans Aging Cohort Study. Pharmacoepidemiol Drug Saf Jul 2011;20(7):689–699. https://doi.org/10.1002/pds.2148.
[23] Jepsen P, Vilstrup H, Lash TL. Development and validation of a comorbidity scoring system for patients with cirrhosis. Gastroenterol Jan 2014;146(1):147–156. https://doi.org/10.1053/j.gastro.2013.09.019. quiz e15-6.
[24] Goldberg DS, Newcomb C, Gilroy R, et al. Increased distance to a liver transplant center is associated with higher mortality for patients with chronic liver failure. Clin Gastroenterol Hepatol Jun 2017;15(6):958–960. https://doi.org/10.1016/j.cgh.2017.02.023.
[25] Andersen R. Nonparametric methods for modeling nonlinearity in regression analysis. Annu Rev Sociol 2009;35:67–85. https://doi.org/10.1146/annurev.soc.34.040507.134631.

[26] Rogal SS, Hansen L, Patel A, et al. AASLD Practice Guidance: palliative care and symptom-based management in decompensated cirrhosis. Hepatol Feb 1 2022. https://doi.org/10.1002/hep.32378.
[27] Ferrell BR, Temel JS, Temin S, et al. Integration of palliative care into standard oncology care: ASCO clinical practice guideline update summary. J Oncol Pract Feb 2017;13(2):119–121. https://doi.org/10.1200/JOP.2016.017897.
[28] Patel K, Tandon P, Hernaez R. Palliative care in the patient with acute-on-chronic liver failure. Clin Liver Dis (Hoboken) May 2022;19(5):198–202. https://doi.org/10.1002/cld.1201.
[29] Hernaez R, Patel A, Jackson LK, et al. Considerations for prognosis, goals of care, and specialty palliative care for hospitalized patients with acute-on-chronic liver failure. Hepatol Sep 2020;72(3):1109–1116. https://doi.org/10.1002/hep.31316.
[30] Wachterman MW, Pilver C, Smith D, et al. Quality of end-of-life care provided to patients with different serious illnesses. JAMA Intern Med Aug 1 2016;176(8):1095–1102. https://doi.org/10.1001/jamainternmed.2016.1200.
[31] Rush B, Walley KR, Celi LA, et al. Palliative care access for hospitalized patients with end-stage liver disease across the United States. Hepatol Nov 2017;66(5):1585–1591. https://doi.org/10.1002/hep.29297.
[32] Sullivan DR, Teno JM, Reinke LF. Evolution of palliative care in the department of veterans affairs: lessons from an integrated health care model. J Palliat Med Jan 2022;25(1):15–20. https://doi.org/10.1089/jpm.2021.0246.
[33] Olmsted CL, Johnson AM, Kaboli P, et al. Use of palliative care and hospice among surgical and medical specialties in the Veterans Health Administration. JAMA Surg Nov 2014;149(11):1169–1175. https://doi.org/10.1001/jamasurg.2014.2101.
[34] Roeland EJ, Triplett DP, Matsuno RK, et al. Patterns of palliative care consultation among elderly patients with cancer. J Natl Compr Canc Netw Apr 2016;14(4):439–445. https://doi.org/10.6004/jnccn.2016.0050.
[35] Wen Y, Jiang C, Koncicki HM, et al. Trends and racial disparities of palliative care use among hospitalized patients with ESKD on dialysis. J Am Soc Nephrol Sep 2019;30(9):1687–1696. https://doi.org/10.1681/ASN.2018121256.
[36] Kathpalia P, Smith A, Lai JC. Underutilization of palliative care services in the liver transplant population. World J Transpl Sep 24 2016;6(3):594–598. https://doi.org/10.5500/wjt.v6.i3.594.
[37] Ufere NN, O'Riordan DL, Bischoff KE, et al. Outcomes of palliative care consultations for hospitalized patients with liver disease. J Pain Symptom Manage Jul 19 2019. https://doi.org/10.1016/j.jpainsymman.2019.07.011.
[38] Ufere NN, Donlan J, Waldman L, et al. Barriers to use of palliative care and advance care planning discussions for patients with end-stage liver disease. Clin Gastroenterol Hepatol Mar 15 2019. https://doi.org/10.1016/j.cgh.2019.03.022.
[39] Esteban JPG, Rein L, Szabo A, et al. Attitudes of liver and palliative care clinicians toward specialist palliative care consultation for patients with end-stage liver disease. J Palliat Med Mar 2019;20. https://doi.org/10.1089/jpm.2018.0553.
[40] Ufere NN, Donlan J, Waldman L, et al. Physicians' perspectives on palliative care for patients with end-stage liver disease: a national survey study. Liver Transpl Jun 2019;25(6):859–869. https://doi.org/10.1002/lt.25469.
[41] Patel AA, Ryan GW, Tisnado D, et al. Deficits in advance care planning for patients with decompensated cirrhosis at liver transplant centers. JAMA Intern Med May 1 2021;181(5):652–660. https://doi.org/10.1001/jamainternmed.2021.0152.
[42] Low J, Davis S, Vickerstaff V, et al. Advanced chronic liver disease in the last year of life: a mixed methods study to understand how care in a specialist liver unit could be improved. BMJ Open Aug 29 2017;7(8):e016887. https://doi.org/10.1136/bmjopen-2017-016887.
[43] Oishi A, Murtagh FE. The challenges of uncertainty and interprofessional collaboration in palliative care for non-cancer patients in the community: a systematic review of views from patients, carers and health-care professionals. Palliat Med Oct 2014;28(9):1081–1098. https://doi.org/10.1177/0269216314531999.
[44] Tandon P, Walling A, Patton H, et al. AGA clinical practice update on palliative care management in cirrhosis: expert review. Clin Gastroenterol Hepatol Apr 2021;19(4):646–656. https://doi.org/10.1016/j.cgh.2020.11.027.
[45] Barnes A, Woodman RJ, Kleinig P, et al. Early palliative care referral in patients with end stage liver disease is associated with reduced resource utilisation. J Gastroenterol Hepatol Oct 15 2019. https://doi.org/10.1111/jgh.14877.
[46] Lee BP, Cullaro G, Vosooghi A, et al. Discordance in categorization of acute-on-chronic liver failure in the United Network for Organ Sharing database. J Hepatol Jan 18 2022. https://doi.org/10.1016/j.jhep.2021.12.040.
[47] Ahluwalia SC, Chen C, Raaen L, et al. A systematic review in support of the national consensus project clinical practice guidelines for quality palliative care. J Pain Symptom Manage Dec 2018;56(6):831–870. https://doi.org/10.1016/j.jpainsymman.2018.09.008.
[48] Goebel JR, Ahluwalia SC, Chong K, et al. Developing an informatics tool to advance supportive care: the veterans health care administration palliative care national clinical template. J Palliat Med Mar 2014;17(3):266–273. https://doi.org/10.1089/jpm.2013.0288.
[49] Foglia MB, Lowery J, Sharpe VA, et al. A comprehensive approach to eliciting, documenting, and honoring patient wishes for care near the end of life: the veterans health administration's life-sustaining treatment decisions initiative. Jt Comm J Qual Patient Saf Jan 2019;45(1):47–56. https://doi.org/10.1016/j.jcjq.2018.04.007.
[50] Naik AD, Arney J, Clark JA, et al. Integrated model for patient-centered advanced liver disease care. Clin Gastroenterol Hepatol May 2020;18(5):1015–1024. https://doi.org/10.1016/j.cgh.2019.07.043.

Journal of Hepatology, Volume *6*

Supplemental information

Rates, patterns, and predictors of specialty palliative care consultation among patients with acute-on-chronic liver failure

Arpan Patel, Anne Walling, Fasiha Kanwal, Marina Serper, Ruben Hernaez, Vinay Sundaram, David Kaplan, Tamar Taddei, and Nadim Mahmud

Rates, patterns, and predictors of specialty palliative care consultation among patients with ACLF

Arpan Patel, Anne Walling, Fasiha Kanwal, Marina Serper, Ruben Hernaez, Vinay Sundaram, David Kaplan, Tamar Taddei, and Nadim Mahmud

Table of contents

Table S1…………………………………………………………………………………..2

Table S2…………………………………………………………………………………..3

Table S3…………………………………………………………………………………..3

Table S4…………………………………………………………………………………..3

Table S5…………………………………………………………………………………..3

Table S6…………………………………………………………………………………..5

Table S7…………………………………………………………………………………..5

Table S8…………………………………………………………………………………..6

Table S1 – Identification of Acute Decompensations (ADs) for EASL-CLIF ACLF

Decompensation	Classification		ICD-9/10 or CPT Codes
**Gastrointestinal Bleed***			
Algorithm: any of the following codes, in any position	UGIB – PUD		531.x, 532.x, 533.x, 534.x; K25.x, K26.x, K27.x, K28.x
	UGIB – non-PUD		456.0, 456.20, 535.x, 537.82, 537.83, 537.84, 578.0; I85.01, I85.11, K29.x, K31.819, K31.811, K31.82, K92.0
	Small bowel GIB		562.0x, 569.84, 569.85, 569.86; K57.1x, K55.20, K55.21, K63.81
	LGIB		562.1x, 569.3; K57.3x, K62.5
	GIB – NOS		578.1, 578.9; K92.1, K92.2
**Infection****			
Algorithm: any of the following codes, in any position	Bacteremia		038.x, 790.7; A40.x, A41.x
	SST/ MSK	Cellulitis	681.x, 682.x; L03.x, K12.2
		Septic arthritis	711.0x excluding 711.08; M00.x
		Osteomyelitis	730.0x, 730.1x, 730.2x; M86.x
	Spontaneous Bacterial Peritonitis		567.23; K65.2
	Pneumonia		481.x, 482.x; J13, J14, J15.x, J18.1, A48.1
	Pyelonephritis/UTI		590.x, 599.0; N10, N11.x, N12, N15.1, N15.9, N16, N28.84, N28.85, N28.86, N39.0
	Other	Endocarditis	421.x; I33.0, I33.9, I39
		Meningitis	320.x; G00.x, G01, G04.2
	Fungal Infection		112.5x, 117.3x, 995.91; B37.7x, B44.9x
**Hepatic Encephalopathy*****			
Algorithm: any of the following codes in any position PLUS lactulose OR rifaximin administered during inpatient admission (see supplemental table 4 for medication identification)	Hepatic encephalopathy		572.2; K72.91
	Viral hepatitis with hepatic coma		070.0, 070.2x, 070.40, 070.41, 070.44, 070.49, 070.6;<br>B15.0, B16.2, B19.11, B16.0, B17.11
**Ascites**			
Algorithm: any of the following codes, in any position	Ascites codes		789.51, 789.59; R18.x
	Paracentesis procedure codes		49080, 49081, 49082, 49083

** Adapted from Abraham et al., Wahl et al., and Siddiqui et al.*
*** Adapted from Schneeweiss et al. and Patkar et al.*
**** Adapted from Lo Re et al.*

Table S2 – Identification of Respiratory Organ Failure
Algorithm: presence of any of the following inpatient CPT codes

ICD-9/10, ICD-9/10-CM, or CPT Codes	Description
31500, 96.05	Endotracheal intubation procedure
94002, 94003, 94004, 94005, 94656, 94657, 94660, 94662, 96.7x, 5A1935Z, 5A1945Z, 5A1955Z	Mechanical ventilation and management
Z99.11	Ventilator dependent status

Table S3 – Identification of Brain Organ Failure
Algorithm: any of the hepatic encephalopathy ICD-9 codes in any position (see supplemental table 1) PLUS lactulose OR rifaximin administered within 28 days of inpatient admission. Identification of these medications was performed as follows:

Medication	SQL Query (VHA database)
Lactulose	WHERE medication LIKE '%LACTULO%' OR medication LIKE '%CONSTULO%' OR medication LIKE '%ENULO%'
Rifaximin	WHERE medication LIKE '%RIFAX%' OR medication LIKE '%XIFAX%'

**Obtained from the "BCMADispensedDrug" table in the VHA CDW; applied within 28 days of admission*

Table S4 – Identification of Inpatient Renal Replacement Therapy
Algorithm: presence of any of the following inpatient CPT codes

CPT Code	Description
90935, 90937	Hemodialysis
90945, 90947	Continuous renal replacement therapy

Table S5 – Identification of Vasopressors for Circulatory Failure

Vasopressors Classified	SQL Query (VHA database)
Norepinephrine<br>Epinephrine<br>Vasopressin	WHERE medication LIKE '%EPINEPHRI%' OR medication LIKE '%VASOPRESSI%' OR medication LIKE '%DOBUTAMIN%' OR

Dobutamine<br>Dopamine<br>Phenylephrine	medication LIKE '%LEVOPHED%' OR medication LIKE '%DOPAMINE%' OR medication LIKE '%PHENYLEPHRIN%'

** Obtained from the "iv_pharmacy_patient" table in the VHA CDW; applied within 28 days of admission*

Table S6 – Mixed-Effects Logistic Regression Models for Late Inpatient Palliative Care Consultation in 90-Day Mortality Subgroup*

Variable	OR (95% CI)	p-value
**Body Mass Index (per 5 unit change)**	1.09 (1.03-1.15)	0.001
**MELD-Na**	0.97 (0.96-0.98)	<0.001
**ACLF Grade**		
1	(ref)	
2	1.42 (1.19-1.68)	<0.001
3	2.07 (1.74-2.48)	<0.001
**Prior Palliative Care Consultation**	0.46 (0.39-0.55)	<0.001
**Hepatocellular Carcinoma**	0.58 (0.49-0.69)	<0.001
**High CIRCOM Score**	0.87 (0.76-0.99)	0.04
**Rural Center Location**	0.47 (0.25-0.88)	0.02

*Mixed effects models, designating VA facility as a random intercept, were used to generate odds ratios and p-values

**Level of significance: *p*-value <0.001

CIRCOM: cirrhosis comorbidity score

Table S7 – Mixed-Effects Logistic Regression Models for Inpatient Palliative Care Consultation, Evaluating the Potential Impact of ACLF Organ Failures*

Variable	OR (95% CI)	p-value
**Age (per year)**	1.03 (1.03-1.04)	<0.001
**Body Mass Index (per 5 unit change)**	0.93 (0.90-0.95)	<0.001
**MELD-Na**	1.01 (1.01-1.02)	<0.001
**Albumin (per 1 unit change)**	0.69 (0.65-0.73)	<0.001
**WBC Count (per 5 unit change)**	1.05 (1.02-1.08)	<0.001
**OF: Kidney**	1.69 (1.54-1.87)	<0.001
**OF: Liver**	2.47 (2.20-2.76)	<0.001
**OF: Coagulation**	1.26 (1.15-1.39)	<0.001
**OF: Brain**	1.36 (1.23-1.50)	<0.001
**OF: Circulatory**	1.82 (1.66-1.99)	<0.001
**Prior Palliative Care Consultation**	2.41 (2.17-2.69)	<0.001
**Hepatocellular Carcinoma**	2.18 (1.95-2.42)	<0.001
**High CIRCOM Score**	0.70 (0.64-0.76)	<0.001
**Cirrhosis Decompensation**	1.17 (1.08-1.27)	<0.001
**Prior TIPS**	0.66 (0.53-0.82)	<0.001

*Mixed effects models, designating VA facility as a random intercept, were used to generate odds ratios and p-values

**Level of significance: *p*-value <0.001

CIRCOM: cirrhosis comorbidity score

Table S8 – Mixed-Effects Logistic Regression Model with Individual CIRCOM Components and Any Cancer History Variable*

Variable	OR (95% CI)	p-value
**Age (per year)**	1.04 (1.03 - 1.04)	<0.001
**Body Mass Index (per 5 unit change)**	0.95 (0.92 - 0.98)	0.001
**MELD-Na**	1.02 (1.02 - 1.03)	<0.001
**Albumin (per 1 unit change)**	0.72 (0.67 - 0.77)	<0.001
**WBC Count (per 5 unit change)**	1.03 (1.00 – 1.07)	0.02
**ACLF Grade**		
1	(ref)	
2	1.74 (1.58 - 1.91)	<0.001
3	3.05 (2.73 - 3.41)	<0.001
**Prior Palliative Care Consultation**	2.62 (2.35 - 2.93)	<0.001
**COPD**	0.86 (0.79 - 0.94)	0.001
**Chronic Kidney Disease**	0.61 (0.56 - 0.66)	<0.001
**Heart Failure**	0.83 (0.76 - 0.91)	<0.001
**Drug Abuse**	1.01 (0.92 - 1.12)	0.78
**Peripheral Artery Disease**	0.82 (0.74 - 0.92)	<0.001
**Epilepsy**	0.88 (0.76 - 1.01)	0.08
**History of Acute MI**	1.19 (0.99 - 1.43)	0.06
**Active MI**	1.02 (0.88 - 1.18)	0.83
**Any Cancer History**	1.42 (1.31 - 1.54)	<0.001
**Cirrhosis Decompensation**	1.15 (1.06 - 1.25)	0.001
**Prior TIPS**	0.64 (0.52 - 0.80)	<0.001

*Mixed effects models, designating VA facility as a random intercept, were used to generate odds ratios and p-values
**Level of significance: *p*-value <0.001
CIRCOM: cirrhosis comorbidity score
